# Multimodal Medical Supervised Image Fusion Method by CNN

**DOI:** 10.3389/fnins.2021.638976

**Published:** 2021-06-02

**Authors:** Yi Li, Junli Zhao, Zhihan Lv, Zhenkuan Pan

**Affiliations:** ^1^College of Data Science Software Engineering, Qingdao University, Qingdao, China; ^2^Business School, Qingdao University, Qingdao, China; ^3^College of Computer Science and Technology, Qingdao University, Qingdao, China

**Keywords:** deep learning, image fusion, CNN, multi-modal medical image, medical diagnostic

## Abstract

This article proposes a multimode medical image fusion with CNN and supervised learning, in order to solve the problem of practical medical diagnosis. It can implement different types of multimodal medical image fusion problems in batch processing mode and can effectively overcome the problem that traditional fusion problems that can only be solved by single and single image fusion. To a certain extent, it greatly improves the fusion effect, image detail clarity, and time efficiency in a new method. The experimental results indicate that the proposed method exhibits state-of-the-art fusion performance in terms of visual quality and a variety of quantitative evaluation criteria. Its medical diagnostic background is wide.

## Introduction

With the continuous recommendation of medical image processing research in recent years, image fusion is an effective solution that automatically detects the information in different images and integrates them to produce one composite image in which all objects of interest are clear. Image fusion (Zhang et al., 2018; Liu et al., 2019; Ma et al., 2019; Pan and Shen, 2019) is a specific algorithm to combine two or more images into a new image. Because of its wide application value, multimodal medical image fusion is an important branch in the field of image fusion. Because of the wide use of multimodal medical images (Yizhang et al., 2019, 2021), this problem has become a hot topic in recent years. At present, in the field of image fusion, deep learning method is a representative method, and it is also one of the research focuses in recent years. Many scholars at home and abroad have conducted on deep learning research and widely applied the research results in image processing and other fields. The recent research in image fusion based on deep learning is as follows: pixel-level image fusion, convolutional neural networks (CNNs) (Liu et al., 2017; Cheng et al., 2018; Hou et al., 2018; Schlemper et al., 2018; Shao and Cai, 2018; Sun et al., 2019), convolutional sparse representation (Li and Wu, 2019), stacked autoencoders (Ahmad et al., 2017; Bao et al., 2017; Jiao et al., 2018; Ma et al., 2018; Chen et al., 2019; Kosiorek et al., 2019), and deep belief network (DBN) (Dong et al., 2016; Chen and Li, 2017; Ye et al., 2018). As an example in CNN research, Shao and Cai (2018) propose a remote sensing image fusion method based on CNN. Cheng et al. (2018) focused on challenges within-class diversity and between-class similarity. The proposed D-CNN models are trained by optimizing a new discriminative objective function. A metric learning regularization term on the CNN features is imposed in this research in order to outperform the existing baseline methods and achieve state-of-the-art results. Level measurement and fusion rule are still important problem in CNN fusion modal. Emphasis in the work of Liu et al. (2017) is laid on these two aspects of research. It proposes a new multifocus image fusion method to overcome the difficulty faced by the existing fusion methods. The method mainly consists of two procedures. First, spatial and spectral features are, respectively, extracted by convolutional layers with different depth. Second, the extracted features from the former step are utilized to yield fused images. In addition, CNN also has a more in-depth study in the field of image action recognition research, image reconstruction. An effective method (Hou et al., 2018) to encode the spatiotemporal information of a skeleton sequence into color texture images is proposed, referred to as skeleton optical spectra, and employs CNNs (ConvNets) to learn the discriminative features for action recognition. This is a typical result of action recognition research. Then, a framework for reconstructing dynamic sequences of two-dimensional cardiac magnetic resonance (MR) images from undersampled data using a deep cascade of CNNs to accelerate the data acquisition process is proposed in research (Schlemper et al., 2018). In addition to the above results, CNN can also be widely used in the study of image super-resolution. In the study of Sun et al. (2019), a novel parallel support vector mechanism (SVM)–based fusion strategy to take full use of deep features at different scales as extracted by the MCNN:Multi-task convolutional neural network structure is proposed. An MCNN structure with different sizes of input patches and kernels is designed to learn multiscale deep features. After that, features at different scales were individually fed into different support vector machine (SVM) classifiers to produce rule images for preclassification. A decision fusion strategy is then applied on the preclassification results based on another SVM classifier. Finally, superpixels are applied to refine the boundary of the fused results using region-based maximum voting.

Furthermore, the more recent theoretical research in the field of deep learning is as follows:

In recent years, deep learning is also widely used in image super-resolution process. In this research, Huang et al.’s 2018 research focused on two defect problems in direction-of-arrival and multiple input multiple output. Deep learning can be applied to solve this problem that resource allocation issue is an obstacle (Takaishi et al., 2018). Importantly, brief advantages of the deep learning based communication schemes are demonstrated in this work. Furthermore, in mobile video research, Liu et al. (2018) point out that the problem of learning temporal dynamics from two aspects is tackled. It is focused on research in the complexity degree of motion among neighboring frames using spatial alignment networks.

The vast majority of these studies focus on the study of single images; the studies of multiple images have been rarely involved. But medical images have specific practical requirements, information richness, and high clarity. Image fusion can increase the amount of information in a single image. To solve this practical medical problem, we propose the method of completion in fusion and hyperscore simultaneously. That is why we are doing this. Multimodal image is a major category in medical image.

In these documents, the requirements of multimodal images for information and clarity have been repeatedly emphasized. In order to effectively meet the needs of the aforementioned medical images and make tentative research on the development of automatic diagnostic technology, supervised deep learning methods were used to achieve image fusion. In this article, deep learning model is intended to be introduced into the field of image fusion. It is intended to develop a new idea of image fusion based on supervised deep learning. We can obtain a new model through the establishment of image training databases in successful fusion results. It is suitable for image fusion to improve the efficiency and accuracy of image process.

## CNN

**FIGURE 1:**
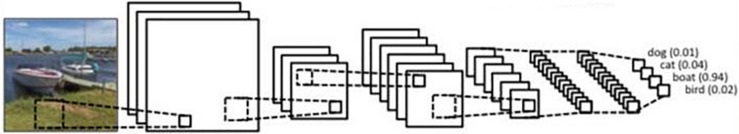
Training process of CNN.

**FIGURE 2:**
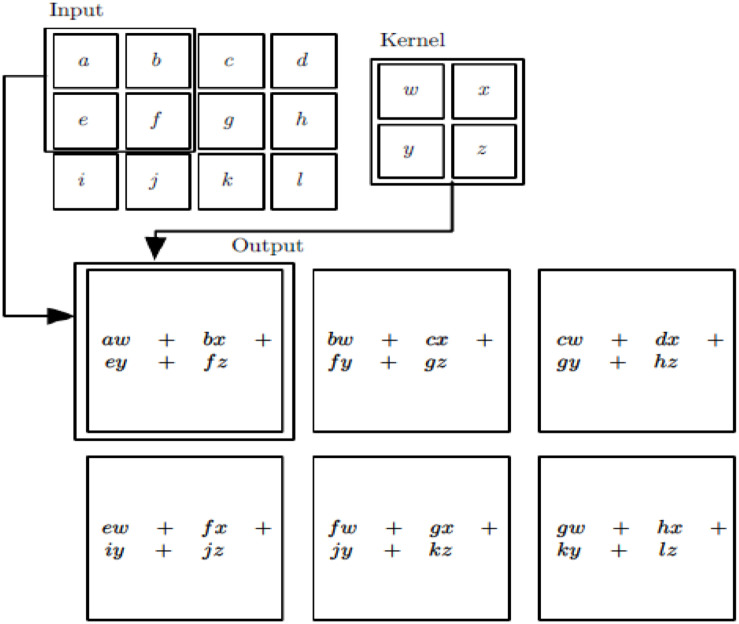
Convolution process.

Deep learning is a hot topic in recent years. Many scholars have focused on their research in several common models such as AutoEncoder (AE), CNN, Restricted Boltzmann Machine (RBM), and DBN. Next, this article summarizes these three models as follows:

As you can see, the image of the ship on the far left is our input layer, which the computer understands as a number of matrices, which is basically the same as DNN. Then, there is the convolution layer, which is specific to CNN, which will be discussed later in (Figure 1). The convolution layer activation function uses a ReLU. We introduced the RELU activation function in DNN, which is very simple ReLU(*x*) = max(0,*x*). Behind the convolution layer is the pooling layer, which is also specific to CNN and will be covered later. Note that there is no activation function for the pooled layer. The convolution formula commonly used here is shown in Formula (1). The detailed convolution process is shown in Figure 2. The third step of the model is to complete the pooling.

(1) $$s(i,j)=(X^{*}W)(i,j)+b=\sum_{k=1}^{n\_in}\left(X_{k}{}^{*}W_{k})(i,j\right)+b$$

## Frames and Algorithms

**FIGURE 3:**
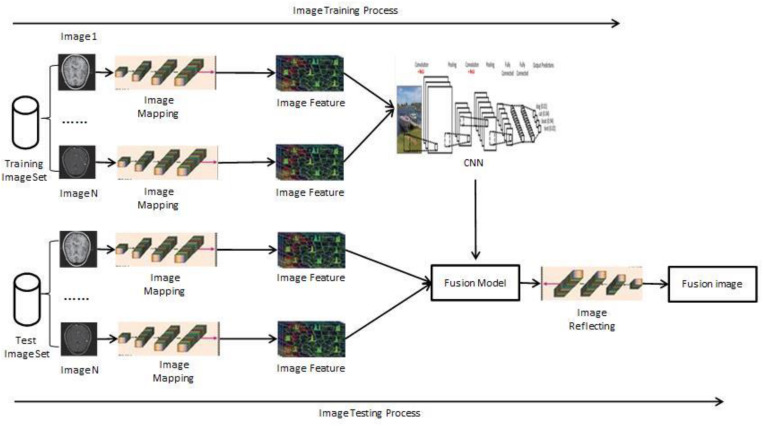
Model of supervised image fusion based on CNN.

The proposed framework is shown in Figure 3. It consists of two major steps: model learning and fusion test. In the first step, the parameters in the DBN model are learned by training the multiple groups of images in the train datasets. Registration processing and pixel alignment in these train images have been achieved in advance. In the second fusion test process, the multiple groups of test images are entered into the model that the learning and training have achieved, and then the fusion process is completed. Next, the final synthesis obtains the fused image.

The new method proposed in this article can realize the fusion task of multimodal medical images.

The specific implementation process is as follows:

Training process:

• In this article, noise removal, registration, standardization, and other preprocessing work will be carried out for a large number of images in datasets. These images will be divided into training datasets and testing datasets in the same level according to the standards of depth learning model.
• The image block size is determined on the training dataset and the test dataset image, respectively. On the two datasets, we use the same block size to complete the model calculation. The size is determined by the standard and the result of the main reference image fusion. If it needs a high precision, it must be selected smaller. If it needs a higher calculation speed, it can be selected larger. In order to better meet the needs of medical diagnosis, different sizes of the calculation model can be used alternately.
• After the training data collection is completed, a fusion calculation model is generated, and related parameters are further optimized. Parameter optimization can be achieved by using automatic optimization.

Testing process:

The final step is also a key step in the process of model testing, in two separate cases. First, the two sets of multimodal images were entered into the fusion model to complete the fusion results. Next, batch processing in multiple group images are achieved. The fusion results were obtained by refactoring, and then these results were output. This model can also complete the process of multi-image fusion at the same time. The specific process is as follows:

• Preprocessed multi-images
• To be fused as input
• Image mapping
• Feature extraction and other work
• Get the fusion results
• Image reconstruction
• To get a fused image

## Experimental and Analysis

Extensive experiments are conducted on different medical image pairs in this part, e.g., computed tomography (CT), MR imaging (MRI), and single-photon emission CT (SPECT). The experimental images are all from public data^1^. The evaluation metrics used in this article are EOG, RMSE, PSNR, ENT, SF, REL, SSIM, H, MI, MEAN, STD, GRAD, *Q*_*0*_, *Q*_*E*_, *Q*_*W*_ and *Q*^*AB*/*F*^; readers can refer to References (Wang and Bovik, 2002; Piella and Heijmans, 2003; Yin et al., 2013; Ma et al., 2015; Liang et al., 2016) for more details. The ranges of *Q*_*0*_, *Q*_*E*_, *Q*_*W*_ and *Q*^*AB*/*F*^ are in [0,1], and the larger the value, the better the fused result. To reduce variation, each experiment is repeated more than 100 times, and their mean values are recorded.

### Databases of Learning

**FIGURE 4:**
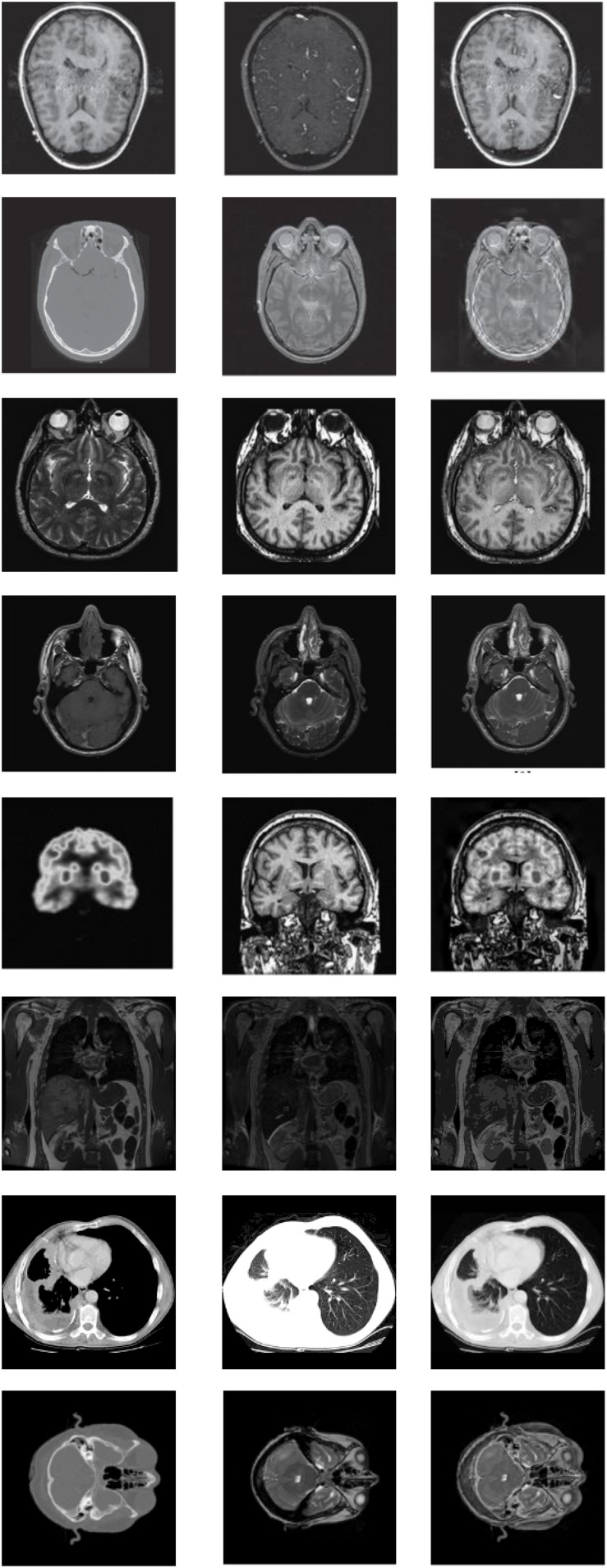
Databases of learning images.

**FIGURE 5:**
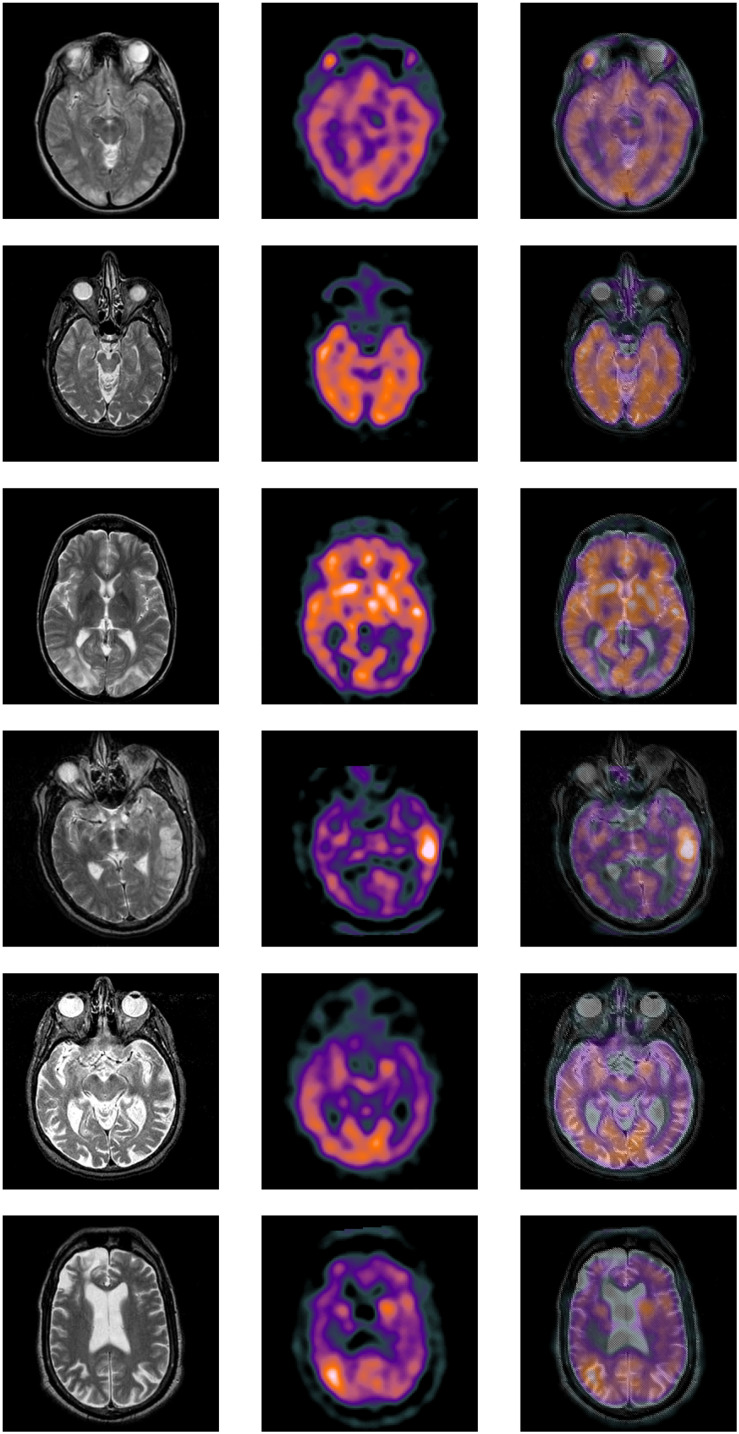
Databases of learning images.

Among the extensive multimodal medical images, the classic images can be divided into two categories: MRI images and CT images. MRI images are more accurate, and its information is more abundant and accurate, especially for human tissue structure and details. CT images provide rich anatomical structure images of the human body. Clinically, images of various anatomical structures can be observed through bone windows, soft tissue windows, and lung windows, and the details of organs can be reflected in detail from a certain angle. SPECT images are one of the typical image types in CT imaging technology. Therefore, the construction of the datasets in the experiment we chose is composited with more classical MRI, CT, and SPECT image in the multimode image. The images were all derived from the standard medical image database and were registered before the experiment. Typical part sets of images are selected to display in this article, as shown in Figures 4, 5. The first image in Figures 4, 5 is an MRI image, the second image is SPECT image, and the third image is a target image.

(1) CT and MRI images data
(2) MRI and SPECT images data

### Experimental Results on CT and SPECT Images

#### Experimental Results of the Proposed Method

**FIGURE 6:**
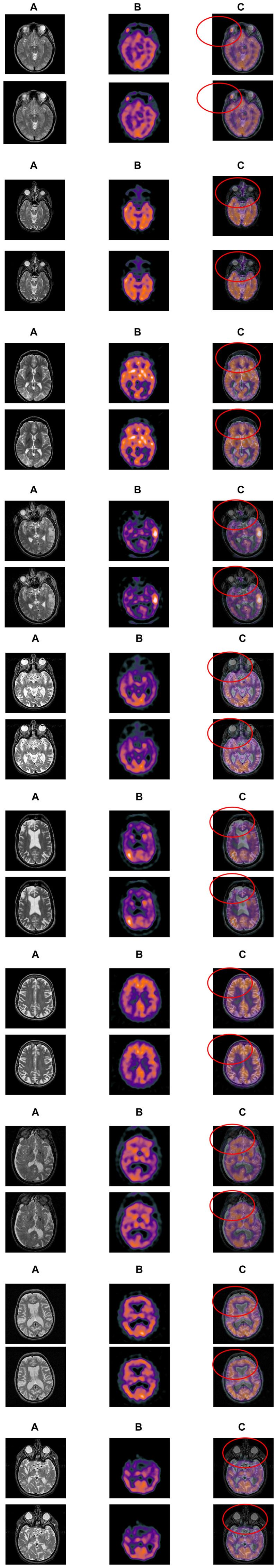
Experiment results on images: **(A)** original image—A; **(B)** original image—B; **(C)** experiment results of image fusion.

**FIGURE 7:**
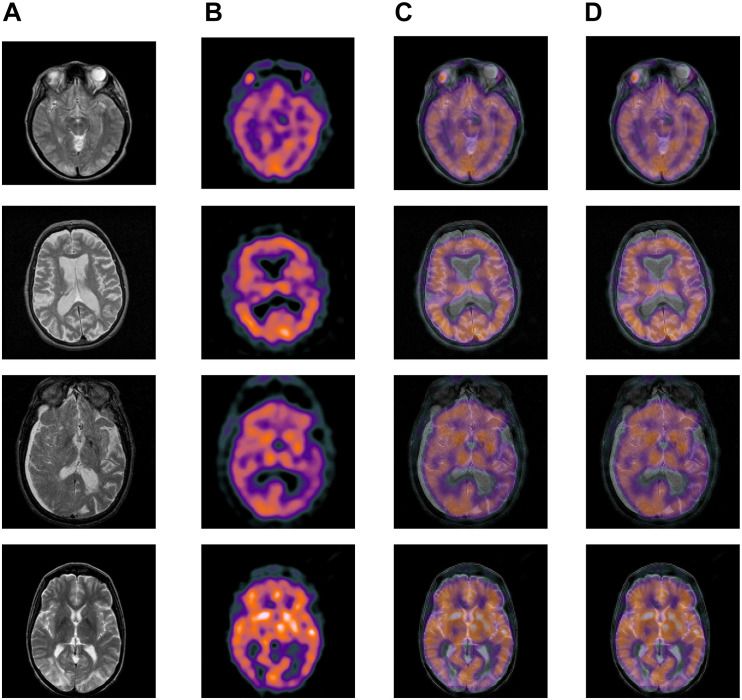
Experiment results on multifocus images: **(A)** original image—A; **(B)** original image—B; **(C)** Liu; (d) Li.

**FIGURE 8:**
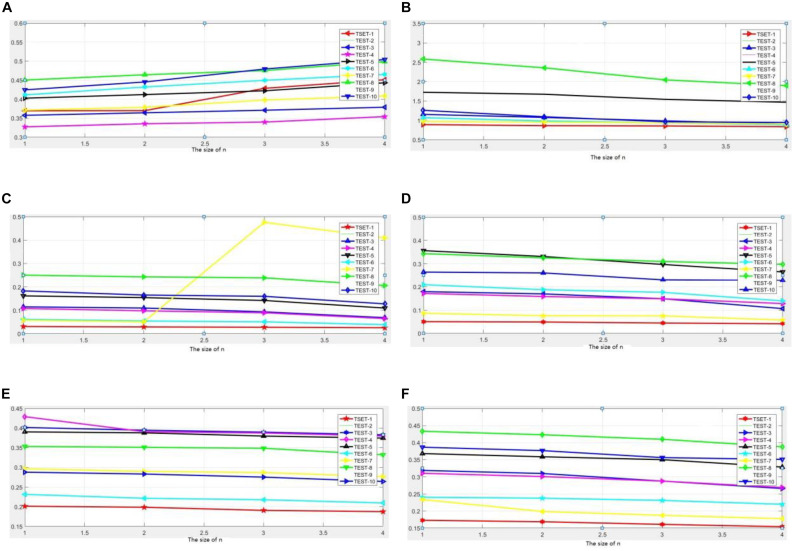
Comparison in block size and fusion performance of the results: **(A)** RMSE, **(B)** entropy, **(C)** MEAN, **(D)** GRAD, **(E)** REL, **(F)** STD.

We have conducted detailed experimental studies on the different disturbance levels of the test images. Here we show the typical two sets of experimental results in the article, as shown in Figures 6, 7 and Tables 1, 2. The perturbation parameters we use are randomly generated; here, we simply refer to “primary disturbance” and “secondary disturbance.” The first set of experimental results in Figure 8 is the result of testing in the case of a primary disturbance, and the second set of experimental results is the result of testing in the case of a secondary disturbance.

**TABLE 1:** Comparison of results.

Indices	Indices											
	
Test images no.	RMSE	PSNR	Entropy	REL	SSIM	H	MI	Mean	STD	ENT	GRAD	Time (s)
Test 1	0.3695	8.6466	0.8918	0.2014	0.9943	0.1987	0.0580	0.0309	0.1730	0.1987	0.0511	1.2776
Test 2	0.3273	9.7007	0.9805	0.4291	0.9955	0.4939	0.1404	0.1080	0.3104	0.4939	0.1718	1.8766
Test 3	0.3576	8.9332	1.1560	0.4016	0.9947	0.5146	0.1483	0.1149	0.3189	0.5146	0.1797	1.9045
Test 4	0.4786	6.4112	2.8396	0.2986	0.9907	0.8361	0.1125	0.2663	0.4420	0.8361	0.3562	1.7547
Test 5	0.4027	7.9010	1.7262	0.3904	0.9932	0.6382	0.1670	0.1617	0.3681	0.6382	0.2093	1.9323
Test 6	0.4113	7.7176	1.0713	0.2315	0.9929	0.3335	0.0893	0.0615	0.2403	0.3335	0.1006	1.5789
Test 7	0.3710	8.6134	0.9708	0.2967	0.9943	0.3194	0.1109	0.0580	0.2337	0.3194	0.0872	1.4625
Test 8	0.4505	6.9269	2.5827	0.3535	0.9916	0.8121	0.1374	0.2505	0.4333	0.8121	0.3433	1.9378
Test 9	0.5499	5.1947	3.1892	0.1770	0.9878	0.9378	0.1259	0.3542	0.4783	0.9378	0.4771	1.6932
Test 10	0.4246	7.4413	1.2606	0.2879	0.9926	0.6866	0.0880	0.1830	0.3867	0.6866	0.2644	1.8334

**TABLE 2:** Comparison of results.

Indices	CT and MRI				MRI and SPECT			
		
Test images no.	Q_0_	Q_*E*_	Q_*W*_	Q^*AB/F*^	Q_0_	Q_*E*_	Q_*W*_	Q^*AB/F*^
Test 1	0.9886	0.8002	0.8707	0.8911	0.7658	0.1177	0.5633	0.2164
Test 2	0.7406	0.3092	0.7411	0.5919	0.7518	0.1578	0.5928	0.2431
Test 3	0.5953	0.1622	0.4622	0.4257	0.7629	0.1363	0.5581	0.2239
Test 4	0.8515	0.3678	0.8870	0.5432	0.6145	0.2247	0.6470	0.2701
Test 5	0.6273	0.2814	0.6935	0.3949	0.6692	0.1350	0.5861	0.2359
Test 6	0.8322	0.5549	0.8716	0.7103	0.7403	0.1392	0.5645	0.2381
Test 7	0.8392	0.3273	0.8057	0.6090	0.7702	0.1511	0.5670	0.2276
Test 8	0.7258	0.3213	0.7667	0.5359	0.6948	0.2668	0.6100	0.2897
Test 9	0.4821	0.2439	0.6412	0.3240	0.4683	0.1519	0.6474	0.3281
Test 10	0.7572	0.2698	0.6946	0.3338	0.5972	0.2298	0.6886	0.2961

#### Comparison With Other Classical Image Fusion Methods Based on Deep Learning

In the experiment, we also carried out this method and compared with the “Li” method in literature (Li and Wu, 2019) and the “Liu” method in literature. The parameters in the model can are obtained by learning, they cannot be determined previously. We have conducted similar experiments on multiple sets of images in the datasets. Here, we present a representative set of experimental results in the article, as shown in Figure 7.

### Analysis of Experimental Results

#### Visual Quality of the Fused Images

First, the visual quality of the fused images obtained by the proposed method is better than the other methods. The fused images obtained by our method look more natural. They produce sharper edges and higher resolution. In addition, the detailed information and interested features are better preserved to some extent, as shown in Figures 6, 7 and Tables 1, 2.

In particular, from the area circled by the red calibration frame, it can be seen that the fusion results are clear, the edges are clear, the information is clearly contrasted, the contrast is obvious, and the key information in the image can be reflected, and the virtual shadow is effectively removed. Moreover, it is clear that the information contained in the fusion image already covers most of the information in the two multimodal images of CT and MRI, MRI and SPECT to be fused, it can be effectively supplementing the deficiencies of the single MRI/CT/SPECT image information. The increase in the amount of information brings changes to the improvement of medical imaging diagnosis undoubtedly. More valuable information can be used to support effective diagnosis. It also brings possibilities for the study of “automatic diagnosis” technology and conducts tentative research. The result of tests 1–10 shown in Figure 6 is the key main similarity between the fusion result and the target image. It can be reflected that the result of the method fusion in this article has covered the main key information of the target image in a more comprehensive way. This can explain the validity of this method further.

#### Analysis of Evaluation Data

From the evaluation data shown in Figure 8, we can find the following:

For the first experiment, the proposed method achieves excellent results when using evaluation metrics :EOG, RMSE, PSNR, ENT, SF, REL, SSIM, H, MI, MEAN, STD, GRAD,_*Q_0_*,*Q_E_*,*Q_W_*_, and _*Q^AB/F^*_. The results show that our methods are effective in image fusion.

Specifically, the result in tests 1–8 of CT and MRI is somewhat better than SR with respect to_*Q_W_*_.

For tests 2–10 experiment results of MRI and SPECT, the proposed method yields outstanding results in terms of_*Q_W_*_, EOG, RESE, PSNR, and REL.

Hence, our method can realize the image fusion task and capture the details of the image compared to other fusion methods.

They results also verify that the process of learning, testing, fusion in this method not only can introduce fusion effectively, but also can make the important information and details in the images integrated by the depth learning model prominently. Fusion result can be covered in key information in two types of medical images, and it can obtain more informative and complete medical image information. However, it is slightly inferior in terms of REL and STD, indicating that the fusion process will cause a loss of information; how to learn model parameters, increase the size of the training data, and improve the degree of training accuracy are our issues for further study.

#### Correlation Between Grid Block Size and Method Validity

**FIGURE 9:**
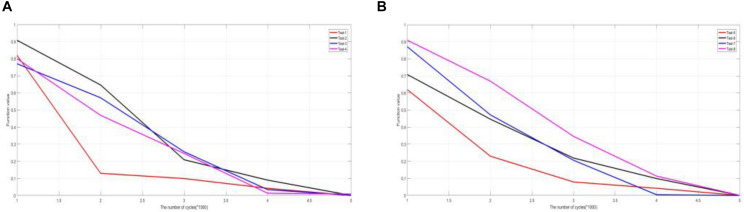
Comparison of experimental results in convergence **(A)** test image 1–4, **(B)** test image 5–8.

In this article, when computing the model learning, we consider small, overlapping image patches instead of direct whole images. In addition, each image is divided into small blocks of size *n* × *n*. Thus, in this section, we intend to explore how the size of the image path affects the fusion performance. Four pairs of source images used in the previous section are utilized in this experiment, and the image is divided into small blocks of size 2*i*(*i* = 0, 1, 2, 3…), in order not to end up with extra grid blocks smaller than the intended size. The larger the value of *i*, the larger the size of the grid block we obtained. For comparison, evaluation metrics EOG, RMSE, PSNR, ENT, SF, REL, SSIM, H, MI, MEAN, STD, GRAD,_*Q_0_*,*Q_E_*,*Q_W_*_, and _*Q^AB/F^*_ are used, and the curves of experimental results are plotted and depicted in Figure 9. The results of the experiment are marked with the mean of 50 operating results in the same environment. The typical six experimental results are presented in the article in Figure 6. We have conducted similar experiments and analyses on other undisplayed indicators and have similar conclusions.

In RMSE, we have conducted detailed experimental studies on the values of *i*(*i* = 1, 2, 3, 4), respectively. Through repeated studies of experimental data, we found that when *i* takes 1 and 2, that is, when the block size takes 2 × 2 and 4 × 4, the error rate of the fusion result is relatively low. When the value *i* gradually climbed to 3 and 4, the error rate increased significantly; the curve gradient increased significantly. This phenomenon has similar conclusions for the 10 sets of images used in testing. It can be explained that selecting different *i* values to achieve the best fusion effect is still different for different fusion images. How to use automatic optimization to dynamically determine the value of *i* need further in-depth research. In terms of image entropy, we also conducted detailed experimental studies on the values of 2*i*(*i* = 1, 2, 3, 4), respectively. Through repeated studies of experimental data, we found that when *i* takes 1 and 2, the information entropy of the fusion results is relatively high. That is to say, the information contained in the fusion image at this time is richer than the result that the block size increases. With the continuous growth of the block size, when the value *i* gradually climbed to 3 or 4, the information entropy was significantly reduced, and along with the increase of *i*, its decline speed increased significantly, and the curve gradient increased significantly. This phenomenon has reached similar conclusions for the 10 groups of images. In particular, in the “test 8” group image experiment, when the value of *i* increased from 2 to 3, the information entropy of the corresponding fusion image accelerated significantly. Similar conclusions can be obtained on the MEAN and GRAD. In the MEAN analysis results, when the value of similar groups of data gradually rises to 3 or 4, the mean decreases significantly.

In the experimental data analysis results of REL and SSIM, we found that there was a difference with the previous four experimental results. In these two indicators, along with the increase of *i*, the REL and SSIM indicators of the fusion image do not appear decline trend significantly, but only slowly slid. It shows that the fusion results obtained by using our fusion method are relatively stable. With the change of the size of the blocks, there is only a small decline. From another aspect, it also shows that our proposed method has a certain degree of stability.

In summary, the determination of the size of the block has a certain influence on the quality of the fusion result, and the small value of *i* will increase the computational workload and time costs inevitably. The better fusion effect can be obtained; this conclusion can be seen from the aspects of error rate, information entropy, mean value, gradient, similarity, etc. Along with the value of *i* continues to grow, the quality of fusion image shows a certain decline in the above indicators, especially *i* takes in Liu et al. (2019), Pan and Shen (2019), this change is even more obvious. This shows that the accuracy of the calculation has decreased with the change of value *i*. As opposed to this, the time cost has decreased. As far as the REL and SSIM similarity metrics are concerned, they are not so sensitive to the changes in the value of *i*, indicating that our proposed method has a certain degree of stability, and this method can be applied to multimodal images effectively. That raises another question that the exact number of *i* that is taken is more appropriate. The exact number of *i* can depend on the specific image fusion problem. The more emphasis on the accuracy of the calculation, the smaller it can be obtained. Conversely, it can be moderately enlarged in order to improve the efficiency of the operation.

#### Method Convergence Analysis

In this section, we take an experimental study on 10 groups of CT and MRI and 10 groups of MRI and SPECT images. The curves of experimental results are plotted and depicted in Figure 9. The results of the experiment are marked with the mean of 50 operating results in the same environment. The typical six experimental results are presented in the article in Figure 9. We have conducted similar experiments and analyses on other undisplayed indicators and have similar conclusions.

A large number of experimental data show that after the number of cycles *n* exceeds 5,000, the function values converge to 0. Function convergence further shows that the method has stability, and there will be no difference in the results in a large number of experiments.

This conclusion can be guaranteed to be accurate and effective in applying this method to medical diagnosis. It is possible that this method is applied in practical medical image processing environment. It also provides an opportunity for further research on the method.

## Conclusion

The application of deep learning techniques to multimodal medical image has been proposed in this article. This article reviews the recent advances achieved in DL image fusion and puts forward some prospects for future study in the field. The primary contributions of this work can be summarized as the following three points.

Deep learning models can extract the most effective features automatically from data to overcome the difficulty of manual design. The method in this article can achieve the multimodal medical image fusion by CNN. Experimental results indicate that the proposed method exhibits state-of-the-art fusion performance in terms of visual quality and a variety of quantitative evaluation criteria. This proposed method can better adapt to the actual needs of medical multimodal image fusion.

In conclusion, the recent research achieved in DL image fusion and super-resolution exhibits a promising trend in the field of image fusion with a huge potential for future improvement. It is highly expected that more related researches would continue in the coming years to promote the development of image fusion.

## Data Availability Statement

The original contributions presented in the study are included in the article/supplementary material, further inquiries can be directed to the corresponding author/s.

## Ethics Statement

Ethical review and approval was not required for the study on human participants in accordance with the local legislation and institutional requirements. Written informed consent for participation was not required for this study in accordance with the national legislation and the institutional requirements.

## Author Contributions

YL was researcher in image processing and pattern recognition, with a major in mathematics and computer science, having plenty of work experience in virtual reality and augmented reality projects, was engaged in the application of computer medical image diagnosis, and her research application fields range widely from deep research fields to everyday lives. All authors contributed to the article and approved the submitted version.

## Conflict of Interest

The authors declare that the research was conducted in the absence of any commercial or financial relationships that could be construed as a potential conflict of interest.

## Footnotes

1 http://www.metapix.de/download.htm#opennewwindow
